# 2-{4-[(2,2-Dimethyl-4,6-dioxo-1,3-dioxan-5-yl­idene)methyl­amino]phen­yl}acetonitrile

**DOI:** 10.1107/S1600536809017437

**Published:** 2009-05-14

**Authors:** Rui Li, Zhen-Yu Ding, Yu-Quan Wei, Jian Ding

**Affiliations:** a State Key Laboratory of Biotherapy, West China Hospital, Sichuan University, Chengdu, 610041, People’s Republic of China; b State Key Laboratory of Drug Research, Shanghai Institute of Materia Medica, Chinese Academy of Sciences, Shanghai, 201203, People’s Republic of China

## Abstract

The title compound, C_15_H_14_N_2_O_4_, is approximately planar, with a dihedral angle of 6.48 (4)° between the amino­methyl­ene unit and the planar five-atom part of the dioxane ring, and a dihedral angle of 2.40 (4)° between amino­methyl­ene unit and the phenyl­ene ring. The dioxane ring is envelope shaped, with the dimethyl-substituted C atom that represents the flap 0.535 (8) Å out of the plane. The mol­ecule has an intra­molecular N—H⋯O hydrogen bond.

## Related literature

For the synthesis of related compounds, see: Cassis *et al.* (1985 ▶). For the synthesis of related anti­tumor precursors, see: Ruchelman *et al.* (2003 ▶). For the crystal structure of a related compound, see: da Silva *et al.* (2006 ▶). For Meldrum’s acid, see: Meldrum (1908 ▶).
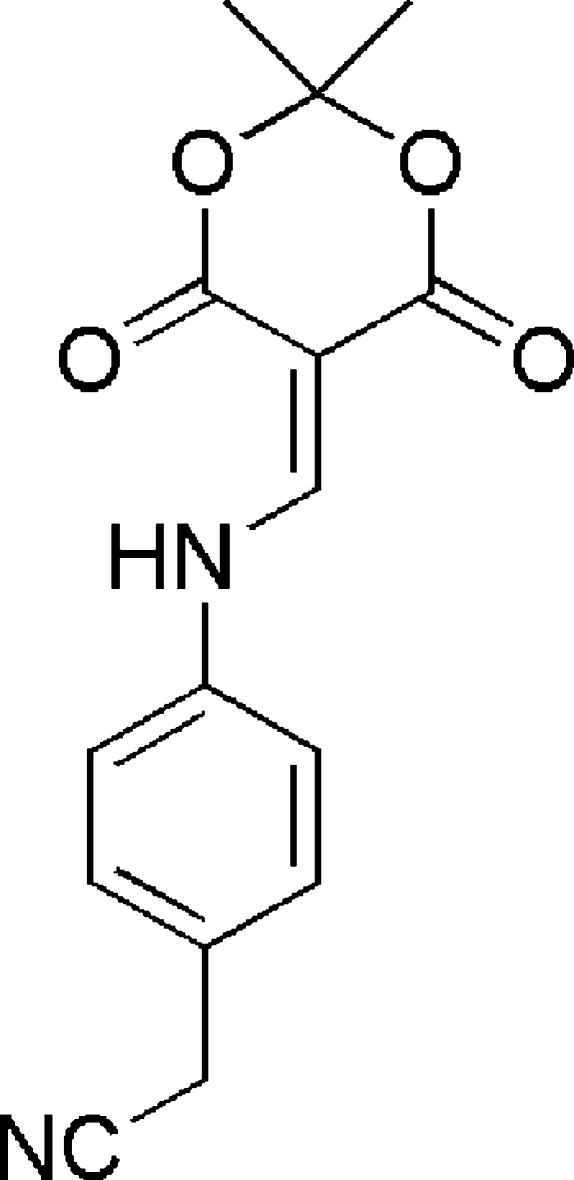


## Experimental

### 

#### Crystal data

C_15_H_14_N_2_O_4_
*M* *_r_* = 286.28
Triclinic, 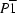

*a* = 5.204 (3) Å
*b* = 11.239 (3) Å
*c* = 12.209 (4) Å
α = 85.51 (3)°
β = 82.30 (3)°
γ = 84.54 (2)°
*V* = 702.9 (5) Å^3^
*Z* = 2
Mo *K*α radiation
μ = 0.10 mm^−1^
*T* = 292 K
0.52 × 0.48 × 0.23 mm

#### Data collection

Enraf–Nonius CAD-4 diffractometer
Absorption correction: none
3217 measured reflections
2609 independent reflections
1610 reflections with *I* > 2σ(*I*)
*R*_int_ = 0.003
3 standard reflections every 150 reflections intensity decay: 1.3%

#### Refinement

*R*[*F* ^2^ > 2σ(*F* ^2^)] = 0.048
*wR*(*F* ^2^) = 0.150
*S* = 1.09
2609 reflections
196 parameters
H atoms treated by a mixture of independent and constrained refinement
Δρ_max_ = 0.19 e Å^−3^
Δρ_min_ = −0.20 e Å^−3^

### 

Data collection: *DIFRAC* (Gabe & White, 1993 ▶); cell refinement: *DIFRAC*; data reduction: *NRCVAX* (Gabe *et al.*, 1989 ▶); program(s) used to solve structure: *SHELXS97* (Sheldrick, 2008 ▶); program(s) used to refine structure: *SHELXL97* (Sheldrick, 2008 ▶); molecular graphics: *ORTEP-3* (Farrugia, 1997 ▶); software used to prepare material for publication: *SHELXL97* and *PLATON* (Spek, 2009 ▶).

## Supplementary Material

Crystal structure: contains datablocks I, New_Global_Publ_Block. DOI: 10.1107/S1600536809017437/ng2579sup1.cif

Structure factors: contains datablocks I. DOI: 10.1107/S1600536809017437/ng2579Isup2.hkl

Additional supplementary materials:  crystallographic information; 3D view; checkCIF report

## Figures and Tables

**Table 1:** Hydrogen-bond geometry (Å, °)

*D*—H⋯*A*	*D*—H	H⋯*A*	*D*⋯*A*	*D*—H⋯*A*
N1—H1n⋯O3	0.97 (2)	1.94 (2)	2.710 (3)	135 (2)

**Fig. 1.**
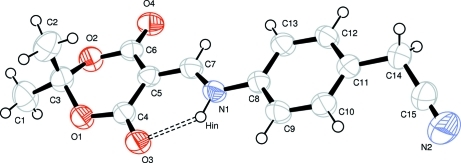
The molecular structure of the title compound, with displacement ellipsoids drawn at the 30% probability level.

**Fig. 2.**
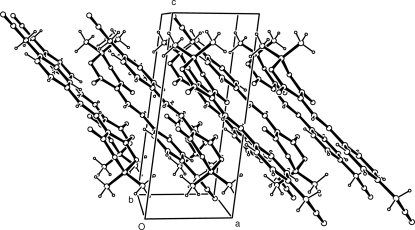
A packing diagram of the title compound showing the layer-like aggregation of the title molecules in the unit cell.

## supplementary crystallographic information

### Comment

The 4(1*H*)quinolone structure plays an extremely important role in the field of pharmaceutical chemistry. These compounds have been used as precursors for anticancer agents, anti-malarial agents and reversible (H+/K+) ATPase inhibitors (Ruchelman *et al.*,2003). 5-arylaminomethylene-2,2-dimethyl-1,3-dioxane-4,6-diones are the key intermediates which can be used to synthesize the 4(1*H*)quinolone derivatives by thermolysis (Cassis *et al.*, 1985).

In the structure of the title molecule (Fig. 1), it is approximately planar with the dihedral angles of 6.48 (4)° and 2.40 (4)° between the connecting aminomethylene unit and the planar part of the dioxane ring, and between the dimethoxybenzyl ring and the aminomethylene group, respectively. Besides, the dioxane ring of the title compound exhibits a half-boat conformation, in which the C atom between the dioxane oxygen atoms is -0.535 (8) Å out-of-plane.

The intramolecular N—H···O hydrogen bond (Table 1) is stabilizing the planar conformation in the molecule. Intermolecular weak C—H···O hydrogen bonding contacts (Table 1) result in the formation of sheets running parallel to the *a-c* plane in the crystal structure (Fig. 2).

### Experimental

A ethanol solution (50 ml) of 2,2-dimethyl-1,3-dioxane-4,6-dione (Meldrum's acid) (1.44 g, 0.01 mol) and methylorthoformate (1.27 g, 0.012 mol) was heated to reflux for 2 h, then the arylamine (1.32 g, 0.01 mol) was added into the above solution. The mixture was heated under reflux for another 8 h and then filtered. Single crystals were obtained from the filtrate after 2 days.

### Refinement

The imino H atom was located in a difference Fourier map and refined isotropically. Other H atoms were positioned geometrically with C—H = 0.93 (aromatic) or 0.96 Å (methyl), and refined using a riding model with *U*_ĩso_(H) = 1.5*U*_eq_(C) for methyl and 1.2*U*_eq_(C) for the others.

### Crystal data

**Table:**

C_15_H_14_N_2_O_4_	*Z* = 2
*M**_r_* = 286.28	*F*(000) = 300
Triclinic, *P*1	*D*_x_ = 1.353 Mg m^−^^3^
*a* = 5.204 (3) Å	Mo *K*α radiation, λ = 0.71073 Å
*b* = 11.239 (3) Å	Cell parameters from 26 reflections
*c* = 12.209 (4) Å	θ = 5.5–9.7°
α = 85.51 (3)°	µ = 0.10 mm^−^^1^
β = 82.30 (3)°	*T* = 292 K
γ = 84.54 (2)°	Block, colourless
*V* = 702.9 (5) Å^3^	0.52 × 0.48 × 0.23 mm

### Data collection

**Table:**

Enraf–Nonius CAD-4 diffractometer	*R*_int_ = 0.003
Radiation source: fine-focus sealed tube	θ_max_ = 25.5°, θ_min_ = 1.7°
graphite	*h* = −6→6
ω/2–θ scans	*k* = −2→13
3217 measured reflections	*l* = −14→14
2609 independent reflections	3 standard reflections every 150 reflections
1610 reflections with *I* > 2σ(*I*)	intensity decay: 1.3%

### Refinement

**Table:**

Refinement on *F*^2^	Primary atom site location: structure-invariant direct methods
Least-squares matrix: full	Secondary atom site location: difference Fourier map
*R*[*F*^2^ > 2σ(*F*^2^)] = 0.048	Hydrogen site location: mixed
*wR*(*F*^2^) = 0.150	H atoms treated by a mixture of independent and constrained refinement
*S* = 1.09	*w* = 1/[σ^2^(*F*_o_^2^) + (0.0853*P*)^2^] where *P* = (*F*_o_^2^ + 2*F*_c_^2^)/3
2609 reflections	(Δ/σ)_max_ < 0.001
196 parameters	Δρ_max_ = 0.19 e Å^−^^3^
0 restraints	Δρ_min_ = −0.20 e Å^−^^3^

### Special details

**Table:**

Geometry. All e.s.d.'s (except the e.s.d. in the dihedral angle between two l.s. planes) are estimated using the full covariance matrix. The cell e.s.d.'s are taken into account individually in the estimation of e.s.d.'s in distances, angles and torsion angles; correlations between e.s.d.'s in cell parameters are only used when they are defined by crystal symmetry. An approximate (isotropic) treatment of cell e.s.d.'s is used for estimating e.s.d.'s involving l.s. planes.
Refinement. Refinement of *F*^2^ against ALL reflections. The weighted *R*-factor *wR* and goodness of fit *S* are based on *F*^2^, conventional *R*-factors *R* are based on *F*, with *F* set to zero for negative *F*^2^. The threshold expression of *F*^2^ > σ(*F*^2^) is used only for calculating *R*-factors(gt) *etc*. and is not relevant to the choice of reflections for refinement. *R*-factors based on *F*^2^ are statistically about twice as large as those based on *F*, and *R*- factors based on ALL data will be even larger.

### Fractional atomic coordinates and isotropic or equivalent isotropic displacement parameters (Å^2^)

**Table:**

	*x*	*y*	*z*	*U*_iso_*/*U*_eq_	
O1	0.5677 (3)	0.03464 (12)	0.29695 (11)	0.0620 (4)	
O2	0.8342 (3)	0.19500 (12)	0.26775 (11)	0.0573 (4)	
O3	0.2465 (3)	0.02806 (12)	0.43304 (11)	0.0639 (4)	
O4	0.7647 (3)	0.35124 (12)	0.36881 (12)	0.0634 (4)	
N1	0.1001 (3)	0.22676 (14)	0.54592 (13)	0.0498 (4)	
H1N	0.074 (5)	0.145 (2)	0.5335 (18)	0.091 (8)*	
N2	−0.9365 (5)	0.3352 (2)	0.9847 (2)	0.1123 (9)	
C1	0.9231 (5)	0.0301 (2)	0.1573 (2)	0.0831 (8)	
H1A	1.0196	0.0754	0.0984	0.125*	
H1B	0.8480	−0.0329	0.1271	0.125*	
H1C	1.0377	−0.0040	0.2090	0.125*	
C2	0.5317 (5)	0.1765 (2)	0.13934 (18)	0.0744 (7)	
H2A	0.3912	0.2203	0.1823	0.112*	
H2B	0.4629	0.1198	0.0986	0.112*	
H2C	0.6268	0.2311	0.0886	0.112*	
C3	0.7107 (4)	0.11099 (19)	0.21537 (16)	0.0573 (6)	
C4	0.4120 (4)	0.08606 (17)	0.38115 (16)	0.0519 (5)	
C5	0.4624 (4)	0.20480 (16)	0.40360 (15)	0.0471 (5)	
C6	0.6901 (4)	0.25836 (17)	0.34787 (15)	0.0477 (5)	
C7	0.3046 (4)	0.26618 (16)	0.48329 (15)	0.0493 (5)	
H7	0.3459	0.3428	0.4941	0.059*	
C8	−0.0785 (4)	0.29056 (16)	0.62235 (15)	0.0472 (5)	
C9	−0.2817 (4)	0.23184 (17)	0.67757 (17)	0.0568 (6)	
H9	−0.2971	0.1528	0.6638	0.068*	
C10	−0.4632 (4)	0.28879 (18)	0.75328 (16)	0.0573 (6)	
H10	−0.5997	0.2476	0.7901	0.069*	
C11	−0.4454 (4)	0.40591 (18)	0.77514 (16)	0.0507 (5)	
C12	−0.2402 (4)	0.46434 (17)	0.71855 (16)	0.0535 (5)	
H12	−0.2253	0.5435	0.7321	0.064*	
C13	−0.0575 (4)	0.40860 (17)	0.64268 (16)	0.0541 (5)	
H13	0.0786	0.4497	0.6055	0.065*	
C14	−0.6446 (4)	0.47201 (19)	0.85500 (16)	0.0608 (6)	
H14A	−0.5548	0.5184	0.8994	0.073*	
H14B	−0.7541	0.5278	0.8130	0.073*	
C15	−0.8082 (5)	0.3952 (2)	0.92822 (19)	0.0722 (7)	

### Atomic displacement parameters (Å^2^)

**Table:**

	*U*^11^	*U*^22^	*U*^33^	*U*^12^	*U*^13^	*U*^23^
O1	0.0652 (9)	0.0508 (8)	0.0660 (9)	−0.0175 (7)	0.0196 (7)	−0.0113 (7)
O2	0.0452 (8)	0.0627 (8)	0.0639 (9)	−0.0173 (7)	0.0036 (7)	−0.0054 (7)
O3	0.0655 (9)	0.0509 (8)	0.0712 (9)	−0.0237 (7)	0.0208 (7)	−0.0066 (7)
O4	0.0655 (10)	0.0527 (8)	0.0744 (9)	−0.0237 (7)	−0.0061 (8)	−0.0008 (7)
N1	0.0521 (10)	0.0400 (9)	0.0560 (9)	−0.0069 (8)	0.0003 (8)	−0.0045 (7)
N2	0.112 (2)	0.0951 (17)	0.1082 (18)	−0.0057 (15)	0.0499 (16)	0.0130 (14)
C1	0.0696 (17)	0.0865 (18)	0.0876 (17)	−0.0170 (15)	0.0277 (14)	−0.0232 (15)
C2	0.0662 (15)	0.0952 (18)	0.0641 (14)	−0.0285 (14)	−0.0041 (12)	−0.0003 (13)
C3	0.0520 (12)	0.0619 (12)	0.0571 (12)	−0.0221 (11)	0.0108 (10)	−0.0073 (10)
C4	0.0511 (12)	0.0481 (11)	0.0539 (11)	−0.0108 (10)	0.0060 (10)	−0.0019 (9)
C5	0.0457 (11)	0.0442 (10)	0.0507 (11)	−0.0097 (9)	−0.0017 (9)	0.0007 (9)
C6	0.0449 (11)	0.0456 (10)	0.0523 (11)	−0.0087 (9)	−0.0041 (9)	0.0016 (9)
C7	0.0522 (12)	0.0413 (10)	0.0537 (11)	−0.0080 (9)	−0.0031 (10)	0.0001 (9)
C8	0.0477 (11)	0.0442 (10)	0.0489 (11)	−0.0069 (9)	−0.0019 (9)	−0.0016 (8)
C9	0.0632 (14)	0.0410 (11)	0.0657 (13)	−0.0151 (10)	0.0017 (11)	−0.0055 (10)
C10	0.0567 (13)	0.0499 (11)	0.0631 (13)	−0.0166 (10)	0.0086 (10)	−0.0036 (10)
C11	0.0506 (12)	0.0497 (11)	0.0501 (11)	−0.0044 (9)	−0.0022 (9)	0.0003 (9)
C12	0.0563 (13)	0.0397 (10)	0.0630 (12)	−0.0061 (9)	0.0018 (10)	−0.0068 (9)
C13	0.0505 (12)	0.0472 (11)	0.0628 (12)	−0.0106 (10)	0.0037 (10)	−0.0029 (9)
C14	0.0619 (14)	0.0592 (12)	0.0572 (12)	−0.0039 (11)	0.0071 (11)	−0.0049 (10)
C15	0.0707 (16)	0.0721 (15)	0.0639 (14)	0.0053 (13)	0.0158 (12)	0.0019 (12)

### Geometric parameters (Å, °)

**Table:**

O1—C4	1.352 (2)	C4—C5	1.438 (2)
O1—C3	1.439 (2)	C5—C7	1.371 (3)
O2—C6	1.356 (2)	C5—C6	1.444 (3)
O2—C3	1.426 (2)	C7—H7	0.9300
O3—C4	1.210 (2)	C8—C9	1.371 (3)
O4—C6	1.205 (2)	C8—C13	1.386 (3)
N1—C7	1.316 (2)	C9—C10	1.379 (3)
N1—C8	1.412 (2)	C9—H9	0.9300
N1—H1n	0.96 (2)	C10—C11	1.378 (3)
N2—C15	1.125 (3)	C10—H10	0.9300
C1—C3	1.500 (3)	C11—C12	1.383 (3)
C1—H1A	0.9600	C11—C14	1.508 (3)
C1—H1B	0.9600	C12—C13	1.377 (3)
C1—H1C	0.9600	C12—H12	0.9300
C2—C3	1.505 (3)	C13—H13	0.9300
C2—H2A	0.9600	C14—C15	1.444 (3)
C2—H2B	0.9600	C14—H14A	0.9700
C2—H2C	0.9600	C14—H14B	0.9700
			
C4—O1—C3	118.27 (15)	O4—C6—C5	125.76 (19)
C6—O2—C3	118.41 (15)	O2—C6—C5	115.95 (15)
C7—N1—C8	127.60 (16)	N1—C7—C5	126.03 (17)
C7—N1—H1N	112.8 (15)	N1—C7—H7	117.0
C8—N1—H1N	119.5 (15)	C5—C7—H7	117.0
C3—C1—H1A	109.5	C9—C8—C13	119.39 (18)
C3—C1—H1B	109.5	C9—C8—N1	117.65 (16)
H1A—C1—H1B	109.5	C13—C8—N1	122.96 (17)
C3—C1—H1C	109.5	C8—C9—C10	120.69 (17)
H1A—C1—H1C	109.5	C8—C9—H9	119.7
H1B—C1—H1C	109.5	C10—C9—H9	119.7
C3—C2—H2A	109.5	C11—C10—C9	120.89 (19)
C3—C2—H2B	109.5	C11—C10—H10	119.6
H2A—C2—H2B	109.5	C9—C10—H10	119.6
C3—C2—H2C	109.5	C10—C11—C12	117.88 (19)
H2A—C2—H2C	109.5	C10—C11—C14	122.30 (19)
H2B—C2—H2C	109.5	C12—C11—C14	119.80 (17)
O2—C3—O1	110.49 (15)	C13—C12—C11	121.86 (17)
O2—C3—C1	106.90 (17)	C13—C12—H12	119.1
O1—C3—C1	105.58 (18)	C11—C12—H12	119.1
O2—C3—C2	109.82 (18)	C12—C13—C8	119.30 (18)
O1—C3—C2	110.28 (17)	C12—C13—H13	120.4
C1—C3—C2	113.65 (19)	C8—C13—H13	120.4
O3—C4—O1	117.85 (16)	C15—C14—C11	114.09 (18)
O3—C4—C5	125.29 (18)	C15—C14—H14A	108.7
O1—C4—C5	116.83 (16)	C11—C14—H14A	108.7
C7—C5—C4	120.65 (17)	C15—C14—H14B	108.7
C7—C5—C6	118.69 (16)	C11—C14—H14B	108.7
C4—C5—C6	120.53 (17)	H14A—C14—H14B	107.6
O4—C6—O2	118.25 (17)	N2—C15—C14	179.5 (3)
			
C6—O2—C3—O1	−49.2 (2)	C8—N1—C7—C5	−174.51 (18)
C6—O2—C3—C1	−163.65 (17)	C4—C5—C7—N1	0.3 (3)
C6—O2—C3—C2	72.6 (2)	C6—C5—C7—N1	−175.62 (18)
C4—O1—C3—O2	46.8 (2)	C7—N1—C8—C9	178.79 (18)
C4—O1—C3—C1	162.08 (18)	C7—N1—C8—C13	−0.7 (3)
C4—O1—C3—C2	−74.8 (2)	C13—C8—C9—C10	−0.4 (3)
C3—O1—C4—O3	162.19 (19)	N1—C8—C9—C10	−179.96 (18)
C3—O1—C4—C5	−19.5 (3)	C8—C9—C10—C11	0.1 (3)
O3—C4—C5—C7	−5.6 (3)	C9—C10—C11—C12	0.2 (3)
O1—C4—C5—C7	176.25 (17)	C9—C10—C11—C14	178.25 (19)
O3—C4—C5—C6	170.2 (2)	C10—C11—C12—C13	−0.1 (3)
O1—C4—C5—C6	−7.9 (3)	C14—C11—C12—C13	−178.27 (19)
C3—O2—C6—O4	−158.33 (18)	C11—C12—C13—C8	−0.2 (3)
C3—O2—C6—C5	23.8 (2)	C9—C8—C13—C12	0.4 (3)
C7—C5—C6—O4	4.2 (3)	N1—C8—C13—C12	179.96 (18)
C4—C5—C6—O4	−171.73 (19)	C10—C11—C14—C15	16.7 (3)
C7—C5—C6—O2	−178.17 (16)	C12—C11—C14—C15	−165.3 (2)
C4—C5—C6—O2	5.9 (3)	C11—C14—C15—N2	−69 (31)

### Hydrogen-bond geometry (Å, °)

**Table:**

*D*—H···*A*	*D*—H	H···*A*	*D*···*A*	*D*—H···*A*
N1—H1n···O3	0.97 (2)	1.94 (2)	2.710 (3)	135 (2)
C7—H7···O4	0.93	2.49	2.816 (3)	100
C9—H9···O3^i^	0.93	2.41	3.292 (3)	159
C13—H13···O4^ii^	0.93	2.51	3.208 (3)	132
C14—H14B···O4^iii^	0.97	2.51	3.343 (3)	143

Symmetry codes: (i) −*x*, −*y*, −*z*+1; (ii) −*x*+1, −*y*+1, −*z*+1; (iii) −*x*, −*y*+1, −*z*+1.
